# Karyotype analyses of ten sections of *Trigonella* (Fabaceae)

**DOI:** 10.3897/compcytogen.v5i2.969

**Published:** 2011-06-01

**Authors:** E. Martin, H. Akan, M. Ekici, Z. Aytac

**Affiliations:** 1 Selçuk University, Faculty of Education, Department of Biology, Konya, Turkey; 2 Harran University, Faculty of Science and Arts, Department of Biology, Şanlıurfa, Turkey; 3 Gazi University, Faculty of Science and Arts, Department of Biology, Ankara, Turkey

**Keywords:** omatic chromosome, Turkey, Fabaceae, karyotype

## Abstract

Karyotypes of ten sections of genus *Trigonella* Linnaeus, 1753 (Fabaceae) from Turkey were investigated. Somatic chromosome numbers of examined species were determined as 2n=14 and 16. The karyotype analyses of the species were carried out and somatic chromosome numbers of *Trigonella plicata* Boiss., 1872, *Trigonella brachycarpa* (Fisch.) Moris, 1833, *Trigonella rostrata* Boiss., 1872, *Trigonella lunata* Boiss., 1843, *Trigonella isthmocarpa* Boiss. et Balansa 1856, *Trigonella rhytidocarpa* Boiss. et Balansa, 1859, *Trigonella spicata* Sibth. et Sm., 1813, *Trigonella cephalotes* Boiss. et Balansa 1856, *Trigonella capitata* Boiss., 1843 and *Trigonella gladiata* Steven, 1808 were reported for the first time. Two pairs of satellite metaphase chromosomes were observed in *Trigonella cariensis* Boiss., 1843 and one pair in *Trigonella lunata*.Moreover, 2 B-chromosomes were found only in *Trigonella procumbens* Rchb., 1830 among all studied species. The aims of this study are to provide karyological data for a significant pool of the taxa, to show differences among them in the number, size and morphology of somatic chromosomes, to verify previous reports or represent numbers which are different from those cited previously.

## Introduction

*Trigonella* L., 1753 (Fabaceae) includes about 135 species worldwide, and most of the species are distributed in dry regions around Mediterranean, West Asia, Europe, North and South Africa, North America, and with only two species being present in South Australia (Mabberly 1997). The genus *Trigonella* has 13 sections and 50 species in Turkey (Huber-Morath 1970). *Trigonella* species are localized in different phytogeographical regions in Turkey with 21 endemic species showing 42% endemism rate (Huber-Morath 1970, Martin et al. 2008).

According to the literature,some studies conducted on the karyology of the *Trigonella* include approximately hundred species (Darlington and Wylie 1955, Tutin and Heywood 1964, Ghosh 1980, Astanova 1981, Agarwal and Gupta 1983, Ladizinsky and Vosa 1986, Danin and Small 1989, Bal 1990, Kumari and Bir 1990, Bidak and Amin 1996, Pavlova 1996, Yılmaz and 2006, Martin et al. 2008). The somatic chromosome numbers of the genus *Trigonella* are reported as 2n=14, 16 and 18. In addition, some chemical, morphological and taxonomical studies were conducted on *Trigonella* species (Meusel and Jager 1962, Sırjaev 1935, Baum 1968, Small et al. 1981, Small et al. 1981, Classen et al. 1982, Small 1988, Danin and Small 1989, Small and Jomphe 1989, Al-Habori et al. 1998, Sheoran et al. 1999, Ram and Verma 2000, Murakami et al. 2000, Oncina et al. 2000, Sur et al. 2001, Sabir et al. 2002, Petropoulos et al. 2002).

In the present work we carried out a karyological study on 19 species of *Trigonella*, belonging to ten sections, collected from different regions of Turkey (Huber-Murath 1970).

## Material and methods

Seedlings were collected between the years of 2002 and 2005 from natural habitats in different localities (Table 1). For karyotype analyses, root tips were obtained from seeds germinated in humidified Petri dishes at room temperature. Root tips were pretreated with α-monobromonaphthalene at 4 °C for 16 h and fixed in Carnoy’s fixative for 24 h at 4 °C. Before staining, the material was hydrolyzed with 1N HCl for 13–15 minutes at room temperature. The chromosomes were stained with 2% aceto orcein and mounted in 45% acetic acid. Permanent slides were made by using the standard liquid nitrogen method and then examined under Olympus BX50 Photomicroscope using an oil immersion objective (100 X). Photographs were taken with the same microscope. Karyotype analyses were made by the use of an Image Analysis System (Bs200Pro).

## Results

This study was carried out to analyse the karyotypes of 19 species, eight of which are endemic to Turkey, belonging to ten sections of the genus *Trigonella* in Turkey. These sections are: *Samaroideae* Boiss., *Pectinatae* Boiss., *Lunatae* Boiss., *Falcatulae* Boiss., *Reflexae* (Širj.) Vass., *Isthmocarpae* Boiss., *Uncinatae* Boiss., *Capitatae* Boiss., *Biebersteinianae* (Širj.), and *Foenum-graecum* Ser. Cytological results obtained from our study are arranged based on the order in the Flora of Turkey (Huber-Morath 1969).

**Figure 1. A–L:**
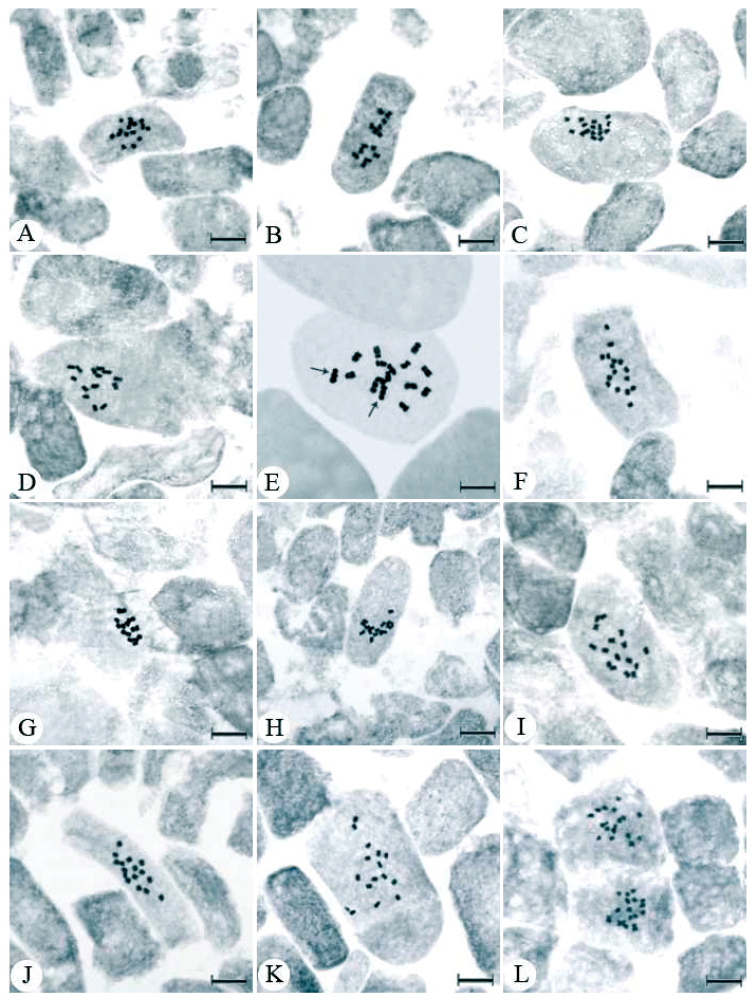
Mitotic metaphase chromosomes of *Trigonella* species **A** *Trigonella cretica* **B** *Trigonella plicata* **C** *Trigonella brachycarpa* **D** *Trigonella rostrata* **E** The satellite (arrow) of chromosomes *Trigonella lunata* **F** *Trigonella corniculata*; no: 4616 **G** *Trigonella corniculata* no: 3391 **H** *Trigonella spinosa* **I** *Trigonella monspeliaca* no: 3358 **J** *Trigonella monspeliaca* no: 3327 **K** *Trigonella isthmocarpa* **L** *Trigonella rhytidocarpa*.Scale bar = 10 µm.

**Figure 1. M–X:**
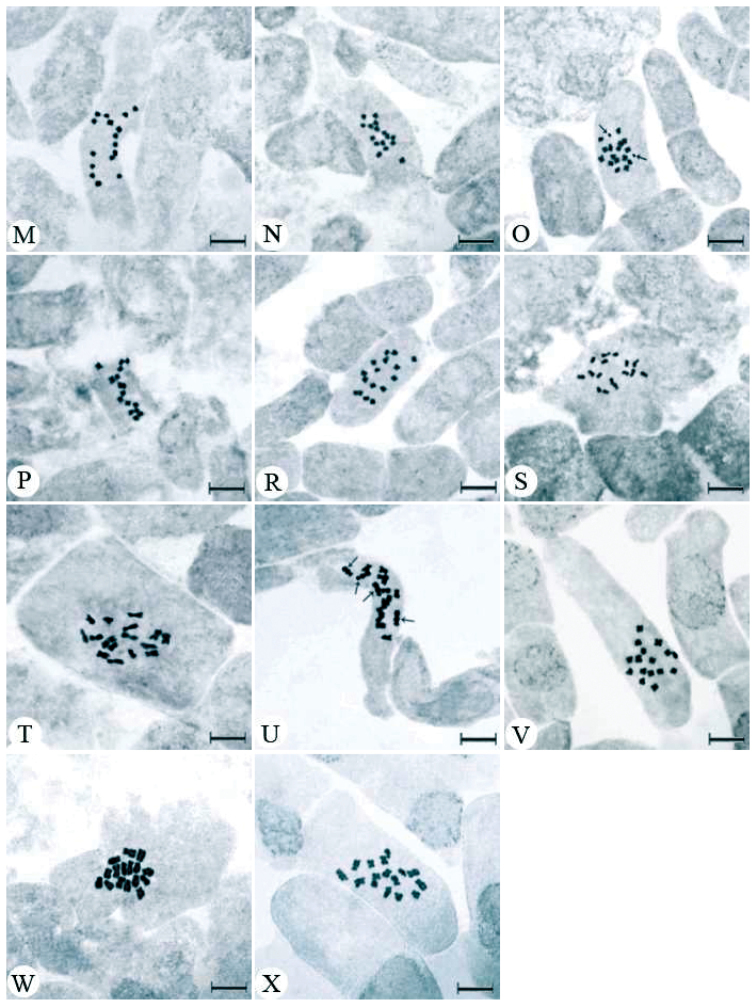
Mitotic metaphase chromosomes of *Trigonella* species **M** *Trigonella spicata* **N** *Trigonella cephalotes* **O** B chromosomes (arrow) *Trigonella procumbens* **P** *Trigonella capitata* **R** *Trigonella coerulescens* no: 3587 **S** *Trigonella coerulescens* no: 3659 **T** *Trigonella gladiata* **U** The satellite (arrow) of chromosomes *Trigonella cariensis* no: 3332 **V** *Trigonella cariensis* no: 4620; **W** *Trigonella foenum-graecum* **X** *Trigonella macrorrhyncha*.Scale bar = 10 µm.

**Figure 2a.**
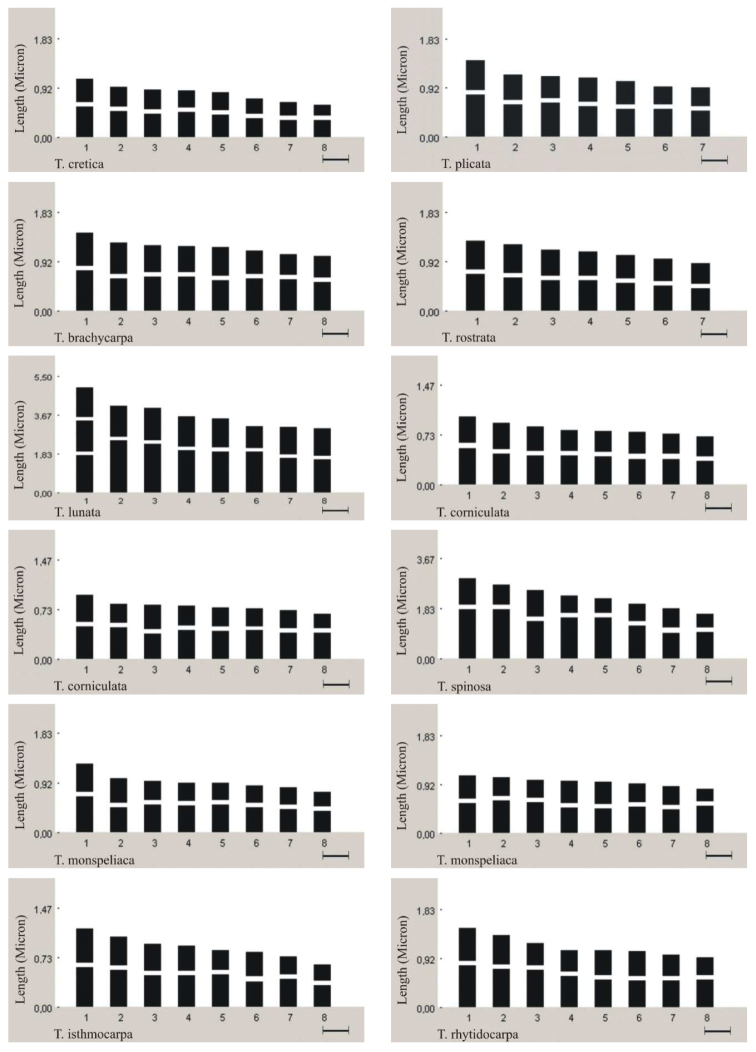
Idiograms of *Trigonella* species (*Trigonella corniculata* no: 4616 and 3391; *Trigonella monspeliaca* no: 3358 and no: 3327; (*Trigonella coerulescens* no: 3587and3659; *Trigonella cariensis* no: 3332 and4620).

**Figure 2b.**
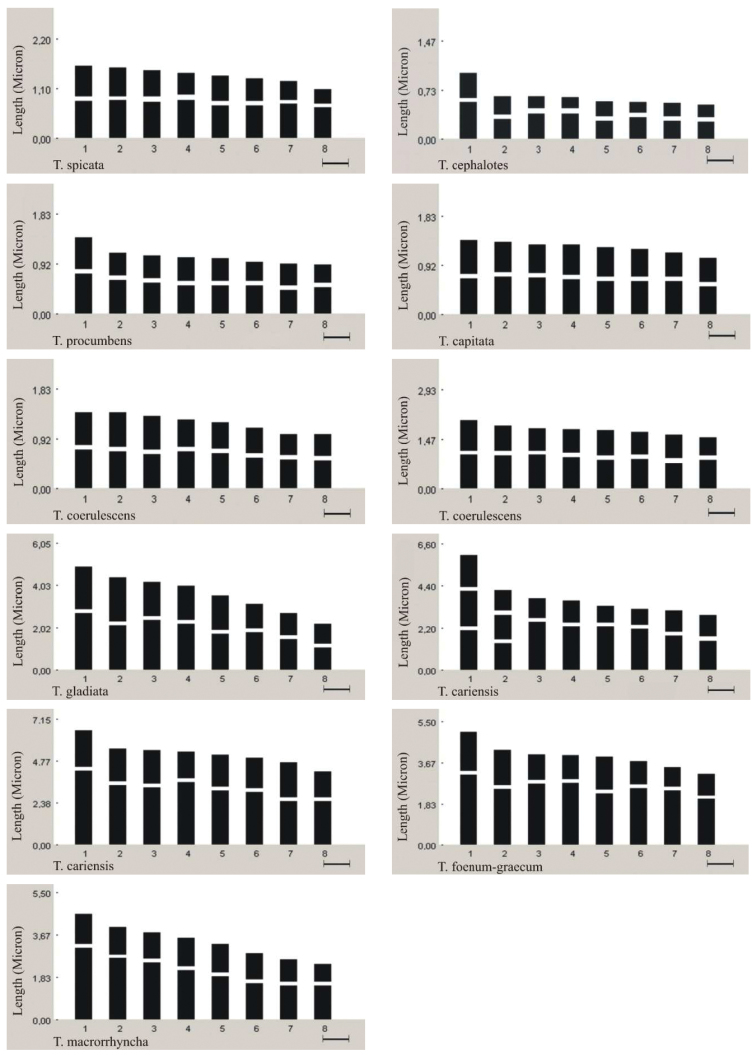
Idiograms of *Trigonella* species (*Trigonella corniculata* no: 4616 and 3391; *Trigonella monspeliaca* no: 3358 and no: 3327; (*Trigonella coerulescens* no: 3587and3659; *Trigonella cariensis* no: 3332 and4620).

**Table 1.** Localities, collector name and numbers of studied *Trigonella* species.

Taxa	Locality	Collector name and number
*Trigonella cretica*	Burdur: Gölhisar-Dirmil 4. km, 30.05.2002, 900–950 m, conserved area	H.Akan 3480, M. Ekici
*Trigonella plicata*	Konya: Hadim-Konya 10. km, 17.07.2002, 1350 m, roadside	H.Akan 3789, Z.Aytaç, M.Ekici
*Trigonella brachycarpa*	Konya: Hadim-Konya 10. km, 17.07.2002, 1400 m, roadside	H.Akan 3786, Z.Aytaç, M.Ekici
*Trigonella rostrata*	Konya: East of Hadim-Karaman, 17.07.2002, 1250 m, roadside	H.Akan 3805, Z.Aytaç, M.Ekici
*Trigonella lunata*	Adana: Pozantı-Çiftehan 8.km, 08.06.2003, 852 m, stony places	H.Akan 4687, M.Ekici
*Trigonella corniculata*	Izmir: Bayraklı, 26.05.2002, 20–50m, meadowsMuğla: Bodrum castle, 25.05.2002, 10 m, meadows	H.Akan 4616, M.EkiciH.Akan 3391, M.Ekici
*Trigonella spinosa*	Muğla: Marmaris-Datça 50. km, 06.05.2005, 5–10 m, maqius	H.Akan 5655, M.Ekici
*Trigonella monspeliaca*	Muğla: Dalaman, Ortaca district, 24.5.2002, 20 m, stony placesAntalya: Exit of Antalya-Hafızpaşa, 23.05.2002, 750 m, maqius	H.Akan 3358, M.EkiciH.Akan 3327, M.Ekici
*Trigonella isthmocarpa*	Aksaray: 28 km E of Aksaray, Hasan mountain foots, 08.06.2003, 1145 m	H.Akan 4695, M.Ekici
*Trigonella rhytidocarpa*	Niğde: Ulukışla-Pozantı 5. km, 16.07.2002, 1300 m, roadside	H.Akan 3778, M.Ekici
*Trigonella spicata*	Antalya-Bucak 20.km, Pınarbaşı district, 23.05.2002, 220–250 m, opening of Quercus coccifera	H.Akan 3319, M.Ekici, Karavelioğulları
*Trigonella cephalotes*	Aydın: Dilek Peninsula, National park, Aydınlık cove, 26.02.2002, 70 m	H.Akan 3398, M.Ekici
*Trigonella procumbens*	Konya: Akşehir-Gelendost 1–2.km, 22.06.2003, 1110 m, roadside of vineyards	H.Akan 4760, M.Ekici
*Trigonella capitata*	Denizli: Pamukkale, 22.06.2003, 1550 m, opening of damaged forest	H.Akan 4767, M.Ekici
*Trigonella coerulescens*	Konya: Ereğli-Karapınar 20–25km, 01.06.2002 1000 m, steppeKayseri: Kayseri, Hisarcık, Erciyes mountain road, 08.07.2002, 1550 m	H.Akan 3587 Z.Aytaç, M.EkiciH.Akan 3659, M.Ekici
*Trigonella gladiata*	Karaman: Pınarbası-Kızılkaya 2 km, 28.06.2005, 1200 m, opening of Oak	H.Akan 5743, M.Ekici
*Trigonella cariensis*	Izmir: Ayrancılar-Izmir 6.km, 25.05.2003, 100 m, foots of maqiusAntalya: Antalya-Hafızpaşa-Bucak 5.km, 23.05.2002, 775 m	H.Akan 4620, M.EkiciH.Akan 3332, M.Ekici
*Trigonella foenum-graecum*	Adana: Ceyhan-Adana 13.km., 20.05.02, 120 m, fields	H.Akan 3274
*Trigonella macrorrhyncha*	Içel: Tarsus-Çamlıyayla road, 30.km, 18.05.2003, 850 m, steppe	H.Akan 4354, M.Ekici

### Section: Samaroideae

*Trigonella cretica* (L.) Boiss., 1872, 2n=16 (Fig. 1, A). All chromosome pairs at the somatic metaphase phase were observed metacentric. The chromosome length ranges between 0.52 and 1.01 μm. The total haploid chromosome length was measured as 5.88 μm.

### Section: Pectinatae

*Trigonella plicata* (Boiss. et Bal.) Boiss., 1872, 2n=14 (Fig. 1, B). The species has metacentric chromosome pairs at somatic metaphase. The chromosome length ranges from 0.84 to 1.34 μm with the total haploid chromosome length of 7.12 μm. This species is endemic of Turkey.

### Section: Lunatae

*Trigonella brachycarpa* (Fisch.) Moris, 1833, 2n=16 (Fig. 1, C). *Trigonella rostrata* (Boiss. & Bal.) Boiss., 1872, 2n=14 (Fig. 1, D). *Trigonella lunata* Boiss., 1843, 2n=16 (Fig. 1, E). All of the species of this section examined have metacentric chromosome pairs at somatic metaphase plates. For *Trigonella brachycarpa*, chromosome length ranges from 0.94 to 1.36 μm having total chromosome length of 8.85 μm. *Trigonella rostrata* being endemic to Turkey was observed having chromosomes whose lengths range between0.81 and 1.22 μm. In this species, the total haploid chromosome length was measured as 7.07 μm. For the species *Trigonella lunata*, the chromosome length was measured between 2.85 and 3.92 μm with the total haploid chromosome length of 26.44 μm. In addition, this species has a pair of satellite metaphase chromosomes.

### Section: Falcatulae

*Trigonella corniculata* L., 1759 from the specimen collected from İzmir province; 2n=16 (Fig. 1, F) and from the specimen collected from Muğla province; 2n=16 (Fig. 1, G). At somatic metaphase, seven pairs of metacentric and one pair of submetacentric chromosomes were observed in the former while four pairs of metacentric and four pairs of submetacenric chromosomes were detected in the latter. The specimen collected from İzmir province has chromosomes ranging between 0.61 and 0.88 μm, and the total haploid chromosome length for this specimen was measured as 5.71 μm. In the other specimen which was collected from Muğla province, the chromosome length ranges between 1.54 and 2.57 μm with the total haploid chromosome length of 17.58 μm. *Trigonella spinosa* L., 1753, 2n=16 (Fig. 1, H). At somatic metaphase, two pairs of metacentric and six pairs of submetacentric chromosomes were observed. For this species, the chromosome lengths range from 1.49 to 2.76 μm with the total haploid chromosome length of 16.76 μm.

### Section: Reflexae

*Trigonella monspeliaca* L. 1753 from the specimen collected from Muğla province; 2n=16 (Fig. 1, I) while the specimen collected from Antalya province; 2n=16 (Fig. 1, J). In the former, all chromosome pairs at the somatic metaphase were observed to be metacentric, and the chromosome length ranges between 0.66 and 1.18 μm with the total haploid chromosome length of 6.81 μm. In the latter specimen, five pairs of metacentric and three pairs of metacentric chromosomes were observed at somatic metaphase. The chromosome length ranges between 0.74 and 1.00 μm. The total haploid chromosome length was measured as 7.06 μm.

### Section: Isthmocarpae

*Trigonella isthmocarpa* Boiss. et Bal., 1856, 2n=16 (Fig. 1, K) and *Trigonella rhytidocarpa* Boiss. & Bal. 2n=16 (Fig. 1, L). Endemic to Turkey, both species have metacentric chromosome pairs at the somatic metaphase. For the species of *Trigonella isthmocarpa*,the chromosome length ranges between 0.57 and 1.10 μm. The total haploid chromosome length was measured as 6.53 μm. For *Trigonella rhytidocarpa*, the chromosome length ranges between 0.85 and 1.40 μm with the total haploid chromosome length of 8.47 μm.

### Section: Uncinatae

*Trigonella spicata* Sibth. et Sm., 1813, 2n=16 (Fig. 1, M) and *Trigonella cephalotes* Boiss. et Bal., 1856, 2n=16 (Fig. 1, N). At somatic metaphase, five pairs of metacentric and three pairs of submetacentric chromosomes were observed for both species. The chromosome length of *Trigonella spicata* ranges between 1.00 and 1.50 μm with the total haploid chromosome length of 10.36 μm. For *Trigonella cephalotes*, the chromosome length ranges between 0.43 and 0.94 μm. The total haploid chromosome length was measured as 4.49 μm. This species is endemic to Turkey.

### Section: Capitatae

*Trigonella procumbens* (Besser) Reichp., 1830, 2n=16 (Fig. 1, O) and *Trigonella capitata* Boiss., 1843, 2n=16 (Fig. 1, P). Both species have metacentric chromosome pairs at the somatic metaphase. Endemic to Turkey, *Trigonella procumbens* have chromosomes ranging from 0.82 to 1.32 μm with the total haploid chromosome length of 7.75 μm. Moreover, two B chromosomes are observed in this species. The other species of the section examined, *Trigonella capitata*, have chromosomes ranging from 0.96 to 1.30 μm. The total haploid chromosome length was measured as 9.33 μm. This species is also endemic to Turkey.

### Section: Biebersteinianae

*Trigonella coerulescens* (Bieb.) Hal., 1901, in the specimen collected from Konya province; 2n=16 (Fig. 1, R) and in the specimen collected from Kayseri province; 2n=16 (Fig. 1, S). All chromosome pairs at the somatic metaphase phase were observed to be metacentric for both specimens. The chromosome length ranges between 1.38 and 1.91 μm for the former while that of the latter ranges between 0.91 and 1.32 μm. The total haploid chromosome length was measured as 12.91 μm in the specimen collected from Konya while that of the other specimen was measured as 9.06 μm.

### Section: Foenum-graecum

*Trigonella gladiata* Stev. Fischer, 1808, 2n=16 (Fig. 1, T). All chromosome pairs at the somatic metaphase phase were observed to be metacentric. The chromosome length ranges between 2.03 and 4.72 μm. The total haploid chromosome length was measured as 27.70 μm. *Trigonella cariensis* Boiss. In the specimen collected from İzmir province; 2n=16 (Fig. 1, U), and in the specimen collected from Antalya province; 2n=16 (Fig. 1, V). Both specimens have four pairs of metacentric and four pairs of submetacentric chromosomes at somatic metaphase. Of the two specimens, the former has chromosomes whose lengths vary from 2.66 to 3.94 μm with the total haploid chromosome length of 25.28 μm. For the latter, the chromosome length ranges between 3.93 and 6.28 μm, and the total haploid chromosome length was measured as 39.78 μm. In addition, one pair of satellite metaphase chromosomes has been detected in the specimen from Antalya province. *Trigonella foenum-graecum* L., 1753, 2n=16 (Fig. 1, W), and *Trigonella macrorrhyncha* Boiss., 1843, 2n=16 (Fig. 1, X). At somatic metaphase, two pairs of metacentric and six pairs of submetacentric chromosomes were observed for both species. For *Trigonella foenum-graecum*, the chromosome length ranges between 3.03 and 4.84 μm, and the total haploid chromosome length was measured as 30.23 μm. Endemic to Turkey, *Trigonella macrorrhyncha* has chromosomes varying from 2.23 to 4.40 μm. In this species, the total haploid chromosome length is 25.67 μm. Idiograms of each species were arranged in order of decreasing length (Fig. 2). The total haploid chromosome lengths were given in Table 2, and the information of the 19 species studied were presented in Table 3.

**Table 2.** Chromosome comparison in the examined species of *Trigonella* (AR: arm ratio; CI: centromeric index; THC: total length of haploid complement; M: metacentric; SM: submetacentric; *one pair of satellites is shown in the chromosome pairs are marked with an asterisk).

Sections and species	Chromosomenumbers (2n=2x)	Chromosomesizes (µm)	AR	CI	THL(µm)	M	SM
Section Samaroideae *Trigonella cretica*	16	0.52–1.01	1.33	5.40	5.88	8	-
Section Pectinatae *Trigonella plicata*	14	0.84–1.34	1.37	6.06	7.12	7	-
Section Lunatae *Trigonella brachycarpa* *Trigonella rostrata* Trigonella lunata*	161416	0.94–1.360.81–1.222.85–3.92	1.261.171.46	5.586.565.12	8.857.0726.44	878	---
Section Falcatulae *Trigonella corniculata* *Trigonella corniculata* *Trigonella spinosa*	161616	0.61–0.881.54–2.571.49–2.76	1.461.781.99	5.154.594.25	5.7117.5816.76	742	146
Section Reflexae *Trigonella monspeliaca* *Trigonella monspeliaca*	1616	0.66–1.180.74–1.00	1.401.50	5.265.11	6.817.06	85	-3
Section Isthmocarpae *Trigonella isthmocarpa* *Trigonella rhytidocarpa*	1616	0.57–1.100.85–1.40	1.311.34	5.475.37	6.538.47	88	--
Section Uncinatae *Trigonella spicata* *Trigonella cephalotes*	1616	1.00–1.500.43–0.94	1.521.59	5.074.96	10.364.49	55	33
Section Capitatae *Trigonella procumbens* *Trigonella capitata*	16+2B16	0.82–1.320.96–1.30	1.311.19	5.435.68	7.759.33	88	--
Section Biebersteinianae *Trigonella coerulescens* *Trigonella coerulescens*	1616	1.38–1.910.91–1.32	1.321.25	5.455.62	12.919.06	88	--
Section Foenum-graecum *Trigonella gladiata* Trigonella cariensis* *Trigonella cariensis* *Trigonella foenum-graecum* *Trigonella macrorrhyncha*	1616161616	2.03–4.722.66–3.943.93–6.283.03–4.842.23–4.40	1.291.831.782.171.85	5.504.594.534.184.37	27.7025.2839.7830.2325.67	84423	-4465

**Table 3.** The information of the 19 species studied.

Section	Species	Chromosome number(2n)	Chromosome numbers reported (2n)	References
Samaroideae	*Trigonella cretica*	16	16	Yılmaz, 2006
Pectinatae	*Trigonella plicata*	14	-	-
Lunatae	*Trigonella brachycarpa*	16	-	-
“	*Trigonella rostrata*	14	-	-
“	*Trigonella lunata*	16	-	-
Falcatulae	*Trigonella corniculata*	16	16	Tutin, Heywood, 1964
“	*Trigonella spinosa*	16	16	Bidak, Amin, 1996
Reflexae	*Trigonella monspeliaca*	16	16	Darlington, Wylie, 1955
Isthmocarpae	*Trigonella isthmocarpa*	16	-	-
“	*Trigonella rhytidocarpa*	16	-	-
Uncinatae	*Trigonella spicata*	16	-	-
“	*Trigonella cephalotes*	16	-	-
Capitatae	*Trigonella procumbens*	16 + 2B	18	Yılmaz, 2006
“	*Trigonella capitata*	16	-	-
Biebersteinianae	*Trigonella coerulescens*	16	16	Yılmaz, 2006
Foenum-graecum	*Trigonella gladiata*	16	16	Bidak, Amin 1996Darlington, Wylie, 1955Ladizinsky, Vosa, 1986
“	*Trigonella cariensis*	16	16	Ladizinsky, Vosa, 1986
“	*Trigonella foenum-graecum*	16	16	Ladizinsky, Vosa 1986; Bal, 1990;Tutin, Heywood, 1964
“	*Trigonella macrorrhyncha*	16	16	Ladizinsky, Vosa, 1986

## Discussion

### 1. Basic chromosome number variations

In this karyological study, two different basic chromosome numbers of x=7 and x=8 were observed in the species belonging to ten sections of *Trigonella*. Bidak and Amin (1996) reported two different basic chromosome numbers of x=8 and x=9 for *Trigonella* species studied. Among studied species, only two species, *Trigonella plicata* and *Trigonella rostrata*, from the sections of *Pectinatae* and *Lunatae*, respectively,have the basic chromosome number of x*=*7.

### 2. Chromosome number and morphology variations

Two different somatic chromosome numbers (2n=14 and 2n=16) were observed in studied sections. The smallest chromosome length is 0.43 μm measured in *Trigonella cephalotes* (section *Uncinatae*) while the biggest of that is 6.28 μm measured in *Trigonella cariensis* (section *Foenum-graecum*). The smallest total haploid chromosome length was measured as4.49 μm from the species of *Trigonella cephalotes* (section *Uncinatae*). *Trigonella cariensis* (section *Foenum-graecum*) has the biggest total haploid chromosome length of 39.78 μm. *Trigonella rostrata* (section *Lunatae*) has the smallest arm ratio (1.17), and *Trigonella foenum-graecum* (section *Foenum-graecum*) has the biggest (2.17). The smallest centromeric index (4.18) was measured in *Trigonella foenum-graecum* (section *Foenum-graecum*) while the biggest of that (6.56) was observed in *Trigonella rostrata* (section *Lunatae*). In this study, there is a marked difference in somatic chromosome lengths compared to other species of the section *Foenum-graecum*. Chromosome numbers are rather close to each other excluding several species (*Lunatae*, *Trigonella corniculata* and *Trigonella spinosa*) in other sections. Besides, 2B chromosome was observed in *Trigonella procumbens* in the section *Capitatae*.

Karyotype formulae of the sections of *Samaroideae*, *Pectinatae*, *Lunatae*, *Isthmocarpae*, *Capitatae* and *Biebersteinianae* are completely composed of metacentric chromosome pairs. Karyotype formulae of species in other sections are composed of metacentric and submetacentric chromosome pairs. While there is one pair of satellite metaphase chromosomes in *Trigonella lunata* (section *Lunatae*), *Trigonella cariensis* (section *Foenum-graecum*) has two pairs of satellite metaphase chromosomes. Section *Samaroideae* is represented by a single species (*Trigonella cretica*) in Turkey. The karyotype of this species has been analysed by Yılmaz (2006) reporting the karyotype formulae 2n=16 as in our study. Our results agree with one of the reports of somatic chromosome number of 2n=16 from the same locality (Yılmaz 2006).

Section *Pectinatae* is represented by a single species (*Trigonella plicata*) in Turkey. Karyotype analysis of this species has been performed by us for the first time. Section *Lunatae* is represented by four species (*Trigonella brachycarpa*, *Trigonella rostrata*, *Trigonella lunata* and *Trigonella sırjaevii* Hub.-Mor., 1939) in Turkey. Making a general evaluation of the section, diploid chromosome numbers were found to be different although the localities of the two types (*Trigonella brachycarpa* and *Trigonella rostrata*) in the section were similar. The diploid chromosome number is 2n=16 in *Trigonella brachycarpa* whereas it is 2n=14 in *Trigonella rostrata*. The smallest chromosome length of 0.81 μm was measured in *Trigonella rostrata* in this section while the biggest of that was observed in *Trigonella lunata* with a length of 3.92 μm. *Trigonella rostrata* is the species with the smallest arm length of 1.17; however, it is also the species with the biggest centromeric index (6.56). *Trigonella lunata* is the one with the biggest total haploid chromosome length of 26.44 μm. This length is rather different compared to the species in other sections. Besides, one pair of satellite chromosome has been observed in *Trigonella lunata*. Karyotype formulae of all species in the section are composed of metacentric chromosome pairs. *Trigonella sırjaevii* could not been studied due to its inability to be germinated.

Section *Falcatulae* is represented by two species (*Trigonella corniculata* and *Trigonella spinosa*) in Turkey. *Trigonella corniculata* was studied in two different localities (İzmir and Muğla). In Muğla case, tetraploidy was observed differing from the other. Total haploid chromosome length was measured as 5.71 μm in İzmir province while that was 17.58 μm in Muğla province. It can be stated that the distinction between the karyological values obtained from these two localities resulted from locality differences. In Izmir province, the karyotype formula was 7m+1sm whereas it was set as 4m+4sm in Muğla province. Our diploid counts are in agreement with the literature such as one of the reports from Turkish material and many others from different territories (Tutin and Heywood 1964). From a karyological point of view, to obtain same results from the same species confirms the previous studies. The species of *Trigonella spinosa* is also placed in the section *Falcatulae*. Compared with the other species (*Trigonella corniculata*) of the section, the chromosome number is the same and the chromosome size measured is very close to each other. However, karyotype formulae are different. Karyotype formulae of *Trigonella spinosa* is 2m+6sm. Bidak et Amin (1996) reported the somatic chromosome numbers as 2n=16 and 18 in *Trigonella gladiata*, 2n=16 in *Trigonella ornithopodiodes*, 2n=16 in *Trigonella spinosa* and 2n=18 in *Trigonella stellata*.

The section *Reflexae* is represented by a single species (*Trigonella monspeliaca*) in Turkey. This species was studied in two different localities. Diploid chromosome numbers are the same (2n=16) in both localities of *Trigonella monspeliaca* species. The chromosome sizes, total haploid chromosome lengths, arm lengths and centromeric indices are very close to each other while the karyotype formulae are different, i.e. the karyotype formula of Muğla province is 8m, of Antalya province is 5m+3sm. For Darlington and Wylie (1955), in a cytological study conducted on species belonging to *Trigonella*, diploid chromosome numbers varied from 2n=16 to 2n=32. For example, they are 2n=16 in *Trigonella gladiata*, 2n=16 in *Trigonella monspeliaca*, 2n=28, 30 and 2n=32 in *Trigonella polyceratia*.

The section *Isthmocarpae* is represented by two species (*Trigonella isthmocarpa* and *Trigonella rhytidocarpa*) in Turkey. Karyology of the two species in this section was studied for the first time. The diploid chromosome number of *Trigonella isthmocarpa* and *Trigonella rhytidocarpa* species were found to be 2n=16=8m. Karyological characteristics of these two species are very close to each other.

The section *Uncinatae* is represented by two species (*Trigonella spicata* and *Trigonella cephalotes*) in Turkey. The diploid chromosome number of *Trigonella spicata* and *Trigonella cephalotes* were found to be 2n=16=5m+3sm. Karyological characteristics of these two species are very close to each other. There is a marked difference only in terms of total haploid chromosome length. While total haploid chromosome length was 10.36 μm in *Trigonella spicata*, that is 4.49 μm in *Trigonella cephalotes*. *Trigonella cephalotes* is also the species having the smallest haploid chromosome length among studied species.

The section *Capitatae* is represented by three species (*Trigonella procumbens*, *Trigonella capitata* and *Trigonella pseudocapitata*) in Turkey. The diploid chromosome number of *Trigonella procumbens* and *Trigonella capitata* was found as 2n=16=8m. Karyological characteristics of these two species are very close to each other. In addition to A chromosomes, two examples of B chromosomes were observed in *Trigonella procumbens* differing from the other species of the sections studied. The chromosome number of *Trigonella procumbens* was reported as 2n=18 by Yılmaz (2006) from the same locality, but he did not mention B chromosomes. The other species of the section, *Trigonella pseudocapitata*,could not be studied due to its inability to germination.

The section *Biebersteinianae* is represented by *Trigonella coerulescens* in Turkey. It was studied from two different localities. Diploid chromosome numbers are the same (2n=16=8m) in both localities of *Trigonella coerulescens*. Chromosome sizes, total haploid chromosome lengths, arm lengths and centromeric indices are very close to each other. The chromosome number of *Trigonella coerulescens* is in agreement with the previous report (2n=16) by Yılmaz (2006).

The section *Foenum-graecum* is represented by five species (*Trigonella gladiata*, *Trigonella cariensis*, *Trigonella foenum-graecum*, *Trigonella macrorrhyncha* and *Trigonella cassia*) in Turkey. Diploid chromosome numbers of the four species studied in this section are the same (2n=16). *Trigonella cassia* could not be studied due to failure to germinate. The chromosome morphologies of the species are very close to each other. The karyotype formula of *Trigonella gladiata* is 8m. In a cytological study performed by Bidak & Amin (1996), diploid chromosome number was found to be 2n=16 and 2n=18 in *Trigonella gladiata*. Researchers reported two different basic chromosome numbers for this species (x=8 and x=9). However, the basic chromosome number in our study was found to be x=8 for this species. In a cytological study conducted on *Trigonella* species, Darlington and Wylie (1955) reported that the diploid chromosome numbers varied from 2n=16 to 2n=32. For example, 2n=16 in *Trigonella gladiata*, 2n=16 in *Trigonella monspeliaca*, 2n=28, 30 and 32 in *Trigonella polyceratia*. Somatic chromosome numbers found in our study are parallel with that report. Two double satellite chromosome pairs were observed in a sample collected from Antalya. The sample collected from İzmir has the biggest total haploid chromosome length (39.78 μm) among all sections. has diploid chromosome number of 2n=16 as in other species in the section. The karyotype formula is 2m+6sm. *Trigonella foenum-graecum* is the species with the biggest arm ratio in all sections (2.17). Chromosome numbers of only two *Trigonella* species were reported in European Flora records, *Trigonella corniculata* 2n=16 and *Trigonella foenum-graecum* 2n=16 (Tutin and Heywood 1964). Our results obtained from this study agree with that report. In a study on karyotype analysis of *Trigonella foeanum-graecum*, somatic chromosome numbers were found to be similar to that of our study (Bal, 1990). The diploid chromosome number of *Trigonella macrorrhyncha* is 2n=16 as in all other species in the section. The karyotype formula is 3m+5sm. In another study conducted in six different species (*Trigonella gladiata*, *Trigonella cariensis*, *Trigonella foenum-graecum*, *Trigonella berythea*, *Trigonella macrorrhyncha* and *Trigonella cassia*) of the section *Foenum-graecum*, diploid chromosome numbers were reported as 2n=16 (Ladizinsky and Vosa, 1986). In this case, our counts agree with the previous study. It is considered that the results obtained from this karyological study have contributed to the taxonomical revision of the genus *Trigonella*.
